# Asiaticoside-Loaded Multifunctional Bioscaffolds for Enhanced Hyperglycemic Wound Healing

**DOI:** 10.3390/biomedicines13020277

**Published:** 2025-01-23

**Authors:** Raniya Razif, Nur Izzah Md Fadilah, Haslina Ahmad, Daniel Looi Qi Hao, Manira Maarof, Mh Busra Fauzi

**Affiliations:** 1 Department of Tissue Engineering and Regenerative Medicine (DTERM), Faculty of Medicine, Universiti Kebangsaan Malaysia, Cheras 56000, Kuala Lumpur, Malaysia; raniyarazif@gmail.com (R.R.); izzahfadilah@ukm.edu.my (N.I.M.F.); manira@ukm.edu.my (M.M.); 2 Advance Bioactive Materials-Cells UKM Research Group, Universiti Kebangsaan Malaysia, Bangi 43600, Selangor, Malaysia; 3 Integrated Chemical Biophysics Research, Universiti Putra Malaysia (UPM), Serdang 43400, Selangor, Malaysia; haslina_ahmad@upm.edu.my; 4 Department of Chemistry, Faculty of Science, Universiti Putra Malaysia (UPM), Serdang 43400, Selangor, Malaysia; 5 My Cytohealth Sdn Bhd, Hive 5, Taman Teknologi, MRANTI, Bukit Jalil 57000, Kuala Lumpur, Malaysia; dr.daniellooi@cytoholdings.com

**Keywords:** asiaticoside, bioscaffolds, hyperglycemic, wound healing, biomaterials, *Centella asiatica*

## Abstract

The review explores the potential of asiaticoside-loaded bioscaffolds to improve the management of hyperglycemic wounds, particularly diabetic foot ulcers (DFUs). Asiaticoside, sourced from *Centella asiatica*, possesses properties that address DFUs’ healing challenges: insufficient angiogenesis, persistent inflammation, and delayed tissue regeneration. By incorporating asiaticoside into bioscaffold 3D designs including hydrogels, microneedle arrays, and nanofibrous meshes, therapeutic efficacy is optimized. This review examines the mechanisms of asiaticoside in wound healing (collagen production, angiogenesis modulation, inflammation reduction, and cell migration and proliferation) based on in vitro and in vivo studies. Asiaticoside also demonstrates synergistic abilities with other biomaterials, creating the possibility of more effective therapies. While preclinical research is promising, clinical trials are crucial to evaluate the efficacy and safety of asiaticoside-loaded bioscaffolds in patients with DFUs. Asiaticoside-loaded bioscaffolds are a significant development in wound healing and may aid in treating hyperglycemic wound complications. Their ability to offer individualized treatment plans has the potential to enhance the quality of life of those who suffer from diabetes. This review is based on a thorough literature search (2019–2024) across multiple databases, excluding secondary literature and non-English articles.

## 1. Introduction

**Figure 1:**
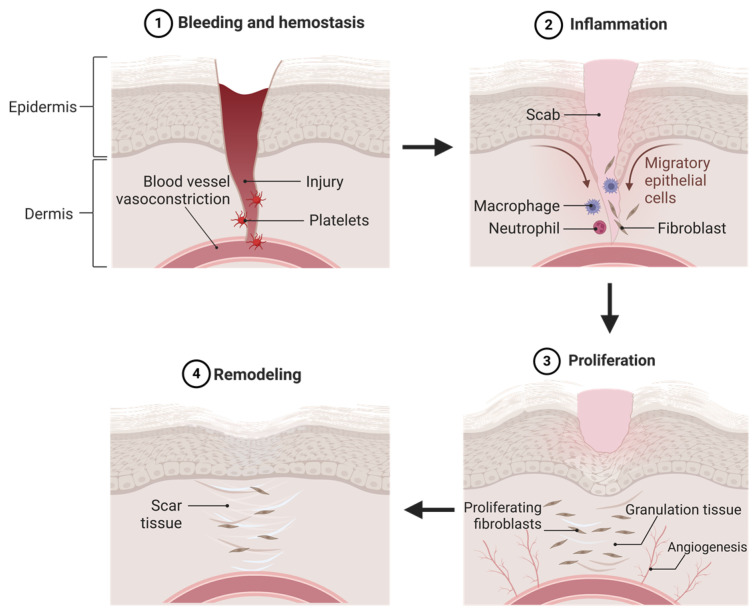
The wound healing phases are (**1**) hemostasis; (**2**) inflammation; (**3**) proliferation; and (**4**) tissue remodeling. Illustration by BioRender.com.

Wound healing is a complex biological process that restores tissue structure and function [1]. It involves four key phases: hemostasis, inflammation, proliferation, and tissue remodeling [1]. Acute wounds typically heal faster and with fewer complications than chronic wounds, which may face issues such as poor blood supply, venous drainage problems, or infections [2,3]. Disruptions to any phase can result in chronic wounds or impede healing [4]. Each phase activates cellular processes contributing to skin regeneration, with inflammation, proliferation, and tissue remodeling often overlapping [5]. Figure 1 illustrates the wound healing stages and skin regeneration process in detail.

The hemostasis phase is the body’s immediate response to halt bleeding after tissue and blood vessel disruption in wound healing. Vasoconstriction initially minimizes blood loss, followed by primary and secondary hemostasis [2]. In primary hemostasis, platelets aggregate at the injury site, interacting with ECM components such as fibronectin, collagen, and factor VIII [6] (Figure 1). Secondary hemostasis activates the coagulation cascade, converting fibrinogen to fibrin, forming a stabilizing fibrin mesh that traps red blood cells and prevents further bleeding [2]. 

Following that, the inflammation phase begins within hours to days as epithelial cells recruit neutrophils, macrophages, and lymphocytes to the wound site in response to infection, initiating phagocytosis as shown in Figure 1 [3]. Neutrophils infiltrate the wound to eliminate pathogens via phagocytosis [1]. After undergoing apoptosis, neutrophils are cleared by macrophages, which also remove debris and secrete inflammatory mediators such as TNF-α, IL-6, and IL-1β to promote tissue repair [6].

Next comes the proliferation phase, which involves tissue regeneration and repair, driven by the activation and proliferation of keratinocytes, fibroblasts, macrophages, and endothelial cells to promote wound closure [6]. Angiogenesis ensures a nutrient supply to the healing tissue (Figure 1). Macrophages clear debris and release signaling molecules to stimulate collagen production, smooth muscle cell migration, and ECM support for re-epithelialization [7]. Wound contraction occurs as actin and myosin in tissue cells pull the edges together, accelerating closure [1].

After re-epithelialization, the healing process transitions to the tissue remodeling phase, focused on refining and strengthening the new tissue. The scab is replaced by a permanent structure as the epidermal layer proliferates (Figure 1) [8]. Though surface healing seems complete, full tissue recovery may take up to two years as collagen fibers reorganize and mature to restore tensile strength [1].

## 2. Hyperglycemic Wound Healing: Challenges in the Diabetic World

### 2.1. Introduction to Diabetes Mellitus and Wound Healing Impairment

As the global population ages, the number of diabetes patients rises annually. In 2019, there were an estimated 135.6 million people aged 65–99 with diabetes worldwide, a number expected to increase to 195.2 million by 2030, marking it as a significant global epidemic of the 21st century. Approximately 6.4% of the diabetic population suffers from diabetic foot ulcers (DFUs) [9]. A hyperglycemic wound is a type of chronic wound that heals differently than normal acute wounds.

**Figure 2:**
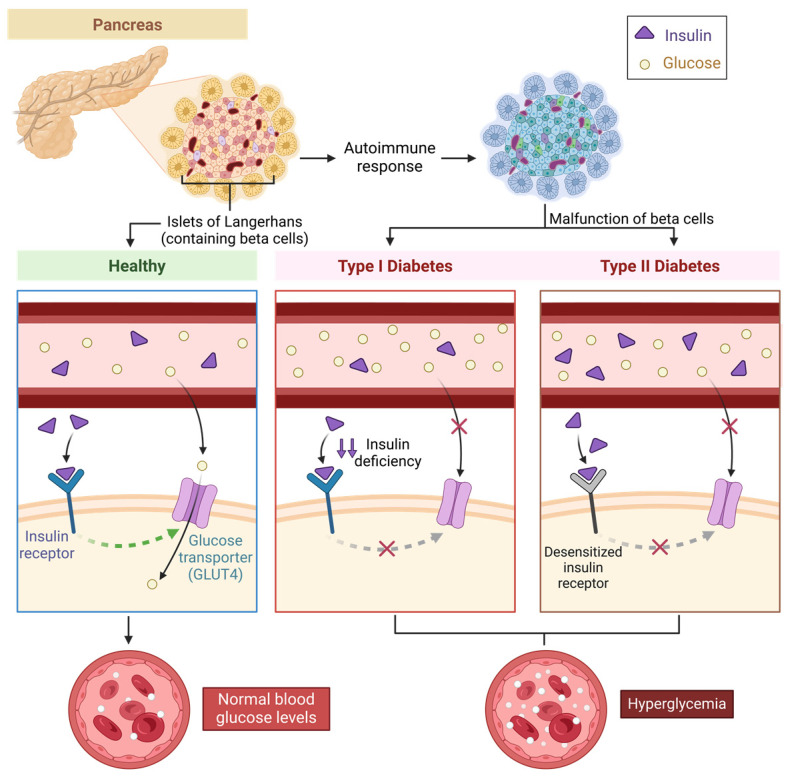
This illustration compares the underlying causes of type 1 and type 2 diabetes. Illustration by BioRender.com.

Type 1 diabetes mellitus (T1DM) is characterized by an autoimmune response that targets and destroys insulin-producing pancreatic beta cells, resulting in insufficient insulin production (Figure 2). This autoimmune disorder can be caused by a complicated interaction of hereditary and environmental factors [10]. The heterogeneity identified in the metabolic, genetic, and immunogenetic profiles of T1DM, as well as age-related changes, highlights the need for specific treatment methods for people with this condition [11]. 

In contrast, type 2 diabetes mellitus (T2DM) is caused by a combination of insulin resistance, where the receptors become desensitized, and beta-cell malfunction (Figure 2). Initially, the body tries to compensate for lower insulin sensitivity by producing more insulin while maintaining normal glucose levels. However, as the disease progresses, beta-cell activity degrades, insulin production decreases, and hyperglycemia develops [12]. T2DM development is complicated, with genetic, environmental, and other risk factors interacting in complex ways. Furthermore, the loss of first-phase insulin release, unusual rapid insulin secretion, and increased glucagon production all contribute to the advancement of T2DM.

Diabetic wounds expose patients to susceptibility of high-risk infection due to incomplete and delayed healing processes in the wounded area. Normally, the affected tissue would start an acute healing process in the wound under normal physiological conditions [13]. It is estimated that one in every three to five patients with diabetes typically develops a chronic non-healing wound such as a diabetic foot ulcer (DFU), a type of wound that has a worrying recurrence rate of 65% within five years and a 40% recurrence rate within one year. The occurrence of DFUs in the overall lifetime of diabetic patients could potentially be as high as 19 to 34% [14].

DFUs have an exceptionally complex pathology due to persistent hyperglycemia and associated diabetic complications, including (1) barrier disruption and infection, (2) high oxidative stress, (3) neuropathy, (4) microvascular complications, and (5) a suboptimal chronic inflammatory response, in addition to psychological problems, including a patient’s mental health, self-esteem, and family cohesion (among others) [14].

### 2.2. Pathophysiology of DFUs

**Figure 3:**
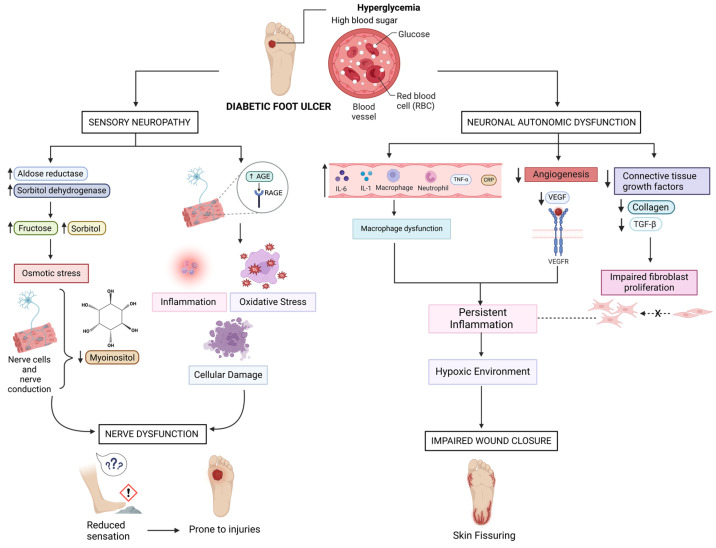
This illustration briefly summarizes a few of the mechanisms involved in DFU healing. Illustration by BioRender.com.

In DFU patients, hyperglycemia upregulates aldose reductase and sorbitol dehydrogenase, increasing fructose and sorbitol production (Figure 3). These products accumulate, causing osmotic stress that reduces myoinositol levels in nerve cells, impairing nerve conduction and leading to sensory deficits (Figure 3). This increases vulnerability to injuries and ulcers due to diminished protective sensation in the feet. Additionally, advanced glycation end products (AGEs) form through non-enzymatic reactions of glucose and dicarbonyls, a process exacerbated in diabetes and linked to complications (Figure 3) [15]. AGEs bind to RAGE receptors on nerve cells, triggering inflammation, oxidative stress, and cellular damage, which contribute to nerve dysfunction (Figure 3). This nerve damage reduces sensation, making patients unaware of minor injuries or pressure points, further increasing ulcer risk.

Diabetes can also cause neuronal autonomic dysfunction in addition to sensory neuropathy, which impairs sweat production and leaves the foot vulnerable to dryness, skin cracking, and fissuring (Figure 3) [16]. Due to an increase in neutrophils, macrophages, and pro-inflammatory cytokines such as interleukin (IL)-1, IL-6, tumor necrosis factor (TNF)-α, and plasma C reactive protein in DFUs, these lesions experience a longer inflammatory phase compared to acute wounds. This persistent inflammation hinders the proliferative phase transition, which is essential for tissue regeneration [14].

In DFUs, impairment of angiogenesis, the formation of new blood vessels, creates a hypoxic environment that hinders wound closure (Figure 3). This is driven by persistent inflammation and reductions in growth factors such as VEGF (Figure 3) [17]. Decreased TGF-β and collagen, due to reduced connective tissue growth factors, further impair fibroblast proliferation and angiogenesis, delaying healing (Figure 3). Additionally, macrophage dysfunction, characterized by elevated TNF and decreased TGF-β1, exacerbates inflammation, reduces collagen production, and prolongs wound closure delays [14].

**Figure 4:**
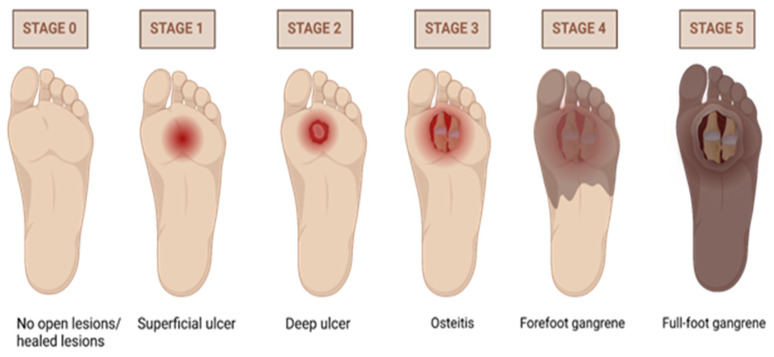
Visual representation of the Wagner grading System for DFUs. Illustration by BioRender.com.

An established framework for evaluating the severity of DFUs is provided by the Wagner grade (Table 1). DFUs are divided into six stages according to the extent of tissue involvement and the existence of infection as shown in Figure 4. This grading system is essential for healthcare professionals, as it helps in determining the appropriate course of treatment, estimating the risk of amputation, and monitoring the progression of the ulcer.

The Wagner grade assists clinicians in making appropriate choices regarding antibiotic therapy, offloading, and wound care. It also monitors patient development and helps healthcare personnel communicate effectively with one another.

## 3. Current Treatment Strategies for DFUs

### 3.1. Conventional DFU Management

DFUs are a major complication of diabetes mellitus, requiring a comprehensive treatment approach due to their complex pathophysiology involving neuropathy, vasculopathy, and infection [19]. Current DFU treatments employ various strategies to address these underlying issues and facilitate wound healing, including traditional methods and newer, innovative treatments. Standard treatment normally includes intensive wound care, debridement, and usage of broad-spectrum antibiotics to control infections, which are typically polymicrobial [20]. Recent innovations have shifted towards fiber-based scaffolds that are enhanced with bioactive compounds to improve healing [20]. Additionally, hydrogels, known for their versatility and hydrophilic properties, have been extensively researched for their potential in drug delivery and tissue engineering applications, showing promising results in DFU treatment [21]. Another example of newer treatment methods would be the usage of hydrogels due to their distinctive properties and therapeutic potential. Hydrogels, with their three-dimensional network structure, offer excellent moisturizing capabilities and permeability and mimic the natural extracellular matrix, which is essential for cell proliferation and wound healing [22]. Various therapeutic modalities have been explored, which include nanomedicine, shockwave therapy, hyperbaric oxygen therapy (HBOT), topical oxygen therapy (TOT), transcutaneous electrical nerve stimulation (TENS), and photobiomodulation (PBM) [23].

### 3.2. Importance of Innovative Treatments for Diabetic Wounds

Diabetic wounds, especially DFUs, pose a major clinical challenge due to the high occurrence rate of DFUs, he prolonged healing process, and the vulnerability of these lesions to major infections significantly impact the quality of life and overall health of diabetic patients [24]. Traditional treatments for diabetic wounds such as antibiotic therapy and surgical debridement face limitations, including the emergence of drug-resistant bacteria and potential side effects from higher drug concentrations [25,26]. Therefore, innovative treatments have become essential for enhancing clinical outcomes. Biomaterial-based wound dressings, for instance, are widely known for their biocompatibility, versatility, and biodegradability, acting as local reservoirs for biomolecules with anti-inflammatory, pro-angiogenic, and antimicrobial properties, thereby enhancing wound healing [27]. Innovative treatments such as hydrogel-based treatment have unique properties, including the ability to create an optimal wound healing environment, reduce infection risks, and retain high moisture; for these reasons, the potential of innovative treatments should be further studied and used in comparison to traditional methods [28]. Another example would be the usage of multifunctional hydrogels that are loaded with bioactive agents such as asiaticoside and recombinant basic human fibroblast growth factor 2 (FGF-2) have demonstrated improved antibacterial, anti-inflammatory, and angiogenesis properties, significantly accelerating the healing of diabetic wounds [29].

This review will examine the emerging field of naturally derived biomaterials such as asiaticoside loaded into bioscaffolds and their potential applications in diabetic wound care.

## 4. Asiaticoside: A Promising Therapeutic Agent

### 4.1. Components of Centella asiatica

**Figure 5:**
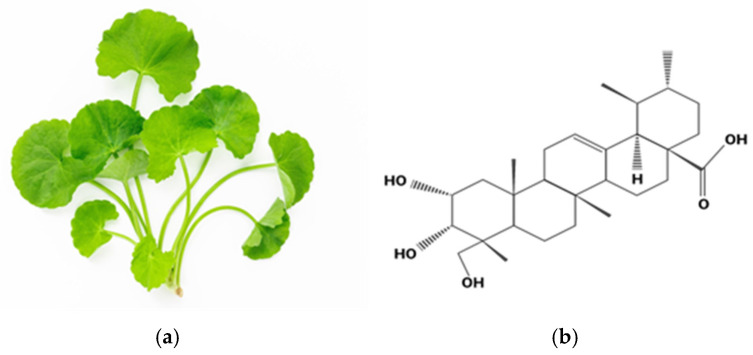
*Centella asiatica* and its bioactive compound. (**a**) Photograph of fresh *Centella asiatica* leaves. Image sourced from *Centella asiatica* stock photos by Vecteezy (https://www.vecteezy.com/free-photos/centella-asiatica), accessed on 30 October 2024. (**b**) Chemical structure of asiaticoside. It is a pentacyclic triterpenoid compound isolated from the plant *Centella asiatica*. The molecule consists of a triterpenoid core and a sugar moiety attached at the C-3 position. The functional groups present in asiaticoside, including hydroxyl and carbonyl groups, contribute to its biological activity. Illustration by BioRender.com.

*Centella asiatica* (Figure 5a), which is also known as gotu kola, is a medicinal plant that is known for its diverse pharmacological properties due to its rich content of bioactive compounds. Research has also demonstrated that the whole-plant extract of *Centella asiatica* is more effective than its chemical components, suggesting a synergistic effect [30]. The main bioactive components of *Centella asiatica* are triterpene ester glycoside compounds which include asiaticoside and madecassoside, and triterpene group compounds, which are madecassic acid (6-hydroxy-asiatic acid), and asiatic acid, responsible for the therapeutic properties and dermatological effects of *Centella asiatica* [31,32,33]. These triterpene compounds in *Centella asiatica* are also known for their neuroprotective, anti-inflammatory, wound-healing, and antioxidant activities; the triterpene compounds can be found in higher concentrations in the aerial parts of the plants, which include leaves and flowers compared, to the stem part of the plant [33,34]. However, the highest antioxidant activity is found in the leaves of the plant compared to the flower and stem of the plant [34].

Asiaticoside presents a compelling choice as the active agent for wound healing due to its dominant presence in *Centella asiatica* [35], suggesting high concentration and potential efficacy. While asiatic acid possesses superior antioxidant activity, asiaticoside can be converted to asiatic acid, potentially offering a two-pronged therapeutic approach [36]. Furthermore, asiaticoside exhibits a wider range of therapeutic benefits, including efficacy in wound healing [36], and demonstrates stronger anti-melanogenesis properties through tyrosinase inhibition and multifaceted influence on melanogenesis pathways [35]. Through this property, asiaticoside would be beneficial in reducing scar pigmentation. Asiaticoside is also known to exhibit anti-inflammatory properties by reducing the levels of pro-inflammatory cytokines such as tumor necrosis factor-alpha (TNF-α) and interleukin-6 (IL-6) [36]. These cytokines are key mediators in the inflammatory process, and their reduction can lead to decreased inflammation. Asiaticoside may aid in alleviating inflammation by suppressing the production of nitric oxide (NO), a signaling molecule [36]. Excessive NO production can intensify inflammatory responses, and by reducing NO levels, asiaticoside plays a role in minimizing inflammation. This combination of dominance, versatility, and synergistic potential with other *Centella asiatica* compounds positions asiaticoside as a promising candidate for wound-healing application.

### 4.2. Asiaticoside and Its Properties for Wound Healing

Asiaticoside is a triterpenoid saponin derived from *Centella asiatica* and is known for its therapeutic capability and extensive pharmacological properties (Figure 5b) [33]. Asiaticoside can demonstrate a wide range of pharmacological effects, such as neuroprotective, cardioprotective, hepatoprotective, wound-healing, anti-inflammatory, antioxidant, anti-allergic, antidepressant, anxiolytic, antifibrotic, antibacterial, anti-arthritic, anti-tumor, and immunomodulatory [37]. However, its therapeutic potential is limited by poor water solubility and low permeability [38]. Structurally, asiaticoside consists of a hydrophobic triterpenoid aglycone and hydrophilic sugar chains (Figure 5b) [39]. It has low biotoxicity and can cross the blood–brain barrier without harmful side effects [40]. Nanocarrier-based delivery systems have been explored to address asiaticoside’s physicochemical limitations and to enhance its efficacy in wound-healing applications [38]. Overall, asiaticoside shows promise as a versatile therapeutic agent, warranting further research to improve its bioavailability and clinical potential.

Specifically for wound healing, asiaticoside has shown high effectiveness in promoting tissue repair and regeneration, which makes asiaticoside very valuable in treating burn-related injuries, ulcers, skin abnormalities, and other related conditions [41]. The anti-inflammatory effects of asiaticoside are well established in various studies. For example, it has been demonstrated to mitigate renal ischemia–reperfusion injury by decreasing the secretion of pro-inflammatory cytokines such as IL-6, IL-1β, and TNF-α while enhancing the anti-inflammatory cytokine IL-10, thereby aiding in tissue repair and reducing inflammation [42]. In the treatment of DFUs, which are chronic and non-healing wounds, asiaticoside has shown considerable effectiveness by stimulating fibroblast proliferation and migration, which is a key factor in wound healing. A study has demonstrated that upregulating Wnt1 and activating the Wnt/β-catenin signaling pathway enhance fibroblast function and expedites wound healing under diabetic conditions [43,44]. It accelerates wound healing by activating the Wnt/β-catenin signaling pathway and increasing the expression of VEGF, iNOS, eNOS, and CD34 [45]. It accelerates wound closure in human gingival fibroblasts and increases Col1A1 gene expression, which is crucial for wound healing [46]. Asiaticoside exhibits various pharmacological activities, including neuroprotective, cardioprotective, and anti-inflammatory effects [37]. In vitro studies on human gingival fibroblasts have shown that asiaticoside accelerates wound healing and increases Col1A1 mRNA expression, suggesting its potential as a wound-healing agent in dentistry [46]. These findings highlight asiaticoside’s diverse mechanisms in promoting wound healing across various cell types and conditions.

## 5. Biomaterials for DFU Treatment

### 5.1. Definition and Types of Bioscaffolds

**Figure 6:**
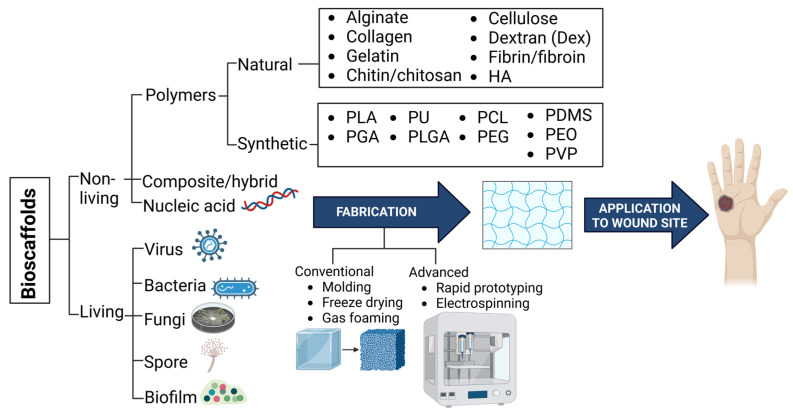
Classification of bioscaffolds, fabrication techniques, and application to wound sites. Illustration by BioRender.com.

In tissue engineering and regenerative medicine, bioscaffolds are synthetic structures that promote the development of new tissues and cell proliferation [47,48]. According to Figure 6, they can be divided into two categories: living (such as bacteria and fungi) and non-living (such as proteins and polymers) [49]. Non-living scaffolds such as natural polymers may resemble natural body structures and are biocompatible and biodegradable; they are beneficial in the healthcare industry. Natural polymers can be generated from sources such as plants and animals. Because of their chemical adaptability, they can be customized to meet different tissue engineering requirements. Additionally, they break down into byproducts that the body can normally absorb. Nonetheless, controlling their rapid degradation can be difficult [50]. While synthetic polymers are stable and have consistent qualities, they do not have the same therapeutic properties as natural polymers. They can be hazardous, but they are usually pure and mechanically robust and break down predictably. These common polymers fall into two categories: hydrophilic (attracting water) and hydrophobic (repelling water) [50]. Nucleic acids (NAs) present an option for the healing of wounds. They can control gene expression and cellular functions, tackling issues such as regulated delivery and maintaining an environment that is conducive to healing. NAs can be used to enhance or repress genes involved in inflammation, cell migration, blood vessel development, and oxidative stress to promote tissue regeneration. These kinds of NAs include siRNA, plasmid DNA, and mRNA [51].

Bioscaffolds offer significant advantages in tissue engineering and wound healing. They provide a three-dimensional structure that mimics the extracellular matrix, supporting cell growth, migration, and differentiation [47,52]. These scaffolds can be designed to incorporate bioactive molecules, growth factors, and cells, enhancing their therapeutic potential [47,53].

### 5.2. Fabrication Techniques for Bioscaffolds

There are various ways to fabricate bioscaffolds, encompassing both conventional and advanced methods (Figure 6). Conventional methods include molding, freeze drying and gas foaming [50]. A typical method for working with thermoplastic polymers is mold fabrication, in which the polymer is melted and then carefully poured into a precisely designed three-dimensional mold [54]. However, the disadvantage of it is that it is unsuitable for hydrogel scaffolds. Removing the biomaterial would probably cause scaffold fracture because it would harm the exterior and interior architecture [55]. Freeze-drying techniques involve casting a polymer solution, freezing it, and subjecting it to a two-step drying process that sublimates ice crystals to form a porous structure. Although pore diameters are not uniform, they can be managed by varying the polymer content, drying time, or temperature [54]. The disadvantage of this method is that it produces a less porous structure than other fabrication methods [50]. By adding gas to a polymer solution—either directly or by in situ generation—conventional gas foaming produces porous scaffolds. Although this process has advantages such as high porosity and mild conditions and does not require organic solvents, it is not as precise in controlling pore size and interconnectivity [56]. It is also worth mentioning that scaffolds fabricated using this method have lower mechanical strength as compared to other methods [50].

Advanced scaffold fabrication techniques, such as rapid prototyping, enable the production of scaffolds that are structurally and mechanically optimized for the specific wound area. These fabrication techniques use a computer-controlled, layer-by-layer additive manufacturing method that enables the precise fabrication of customized 3D scaffolds for tissue engineering. Examples of rapid prototyping bioscaffold fabrication methods include multiphase jet solidification (MJS), 3D plotting, and precise extrusion manufacturing (PEM) [55,57]. Another example of an advanced fabrication method is electrospinning. Electrospinning can be defined as a spinning method using electrostatic forces to create fibrous scaffolds from biocompatible polymers. This method allows the fabrication of fibrous scaffolds that closely resemble the native ECM’s architecture [58]. Unlike conventional fabrication methods that require polymerization outside of the wound site, in situ photopolymerization allows scaffolds to be created in real time, directly in the wound environment [50]. In situ photopolymerization is the process of using light to initiate the transformation of a liquid monomer solution into a solid, cross-linked polymer network [59].

### 5.3. Clinically Available Bioscaffolds

Recent advancements in tissue engineering have led to the development of several clinically viable scaffolds, offering new possibilities for regenerative medicine [60]. One of the types of commercially available bioscaffolds consists of engineered skin substitutes (ESSs). Commercially available skin substitutes, including autologous grafts, allogeneic grafts (e.g., OrCel^®^), engineered substitutes (e.g., Amarantus Bioscience), and cultured epithelial autografts (CEA), present various limitations such as scarring, immune rejection, limited cell survival, and challenges in achieving optimal integration and functionality [45]. There are also commercially available wound-healing scaffolds, such as Integra^®^, Apligraf^®^, and Dermagraft^®^, which have limitations including the need for additional procedures, short shelf life, and limited effectiveness in certain cases. These limitations drive the ongoing development of new scaffolds using advanced technologies such as electrospinning, 3D bioprinting, and microfluidics to create a more physiologically relevant microenvironment for effective wound healing [52].

### 5.4. Rationale for Incorporating Asiaticoside into Bioscaffolds

Integrating asiaticoside into bioscaffolds offers various therapeutic benefits by leveraging its wide range of biological activities to improve tissue regeneration and repair, which includes chronic and complex wounds such as DFUs. Asiaticoside has shown strong capabilities in wound healing, anti-oxidation, and anti-inflammatory activities, which makes it a strong candidate for improving bioscaffolds in tissue engineering and regenerative medicine [33,34,37]. Its wound healing abilities are well established both in vitro and in vivo, as it aids in restoring skin integrity and repairing injured tissues through multiple cellular and biochemical processes, such as boosting collagen synthesis, aiding in cell migration, and reducing oxidative stress [41,61,62]. Furthermore, integrating asiaticoside into different types of bioscaffolds such as hydrogels, nanofibers, and solid lipid nanoparticles will be able to create optimal healing conditions through well-maintained moisture, sustained drug release, and enhanced cellular activities which are essential for tissue regeneration [61]. For example, nanofibrous scaffolds made from polyvinyl alcohol (PVA), sodium alginate (SA), and silk fibroin (SF) and loaded with asiaticoside have shown significant wound-healing effectiveness in diabetic rats. These scaffolds exhibit low cytotoxicity, excellent antimicrobial properties, and the ability to restore normal skin structure [61]. Similarly, chitosan-based hydrogels optimized for delivering asiaticoside have demonstrated promising outcomes in inhibiting hyaluronidase, providing antimicrobial activity, and promoting wound healing through sustained release and efficient skin permeability [63]. Apart from asiaticoside, other plant-based phytoconstituents also exhibit wound-healing capabilities through various properties, including regenerative potential, antioxidant activity, and anti-inflammatory effects. Curcumin, a phenolic compound specifically classified as a polyphenol and found in Curcuma longa (turmeric), is another phytoconstituent with significant potential for chronic wound healing due to its anti-inflammatory, antioxidant, and antimicrobial properties [64,65]. These properties enable curcumin to reduce inflammation and oxidative stress, both of which are critical in the wound-healing process, thereby promoting skin regeneration [66].

Despite its regenerative potential, curcumin’s clinical applications face challenges due to its low water solubility and poor bioavailability. These limitations necessitate the incorporation of additional materials or delivery systems for effective use in both in vivo and in vitro studies [67]. Consequently, asiaticoside emerges as a better choice than curcumin for incorporation into bioscaffolds, as it offers higher bioavailability and can be easily integrated into various types of bioscaffolds.

Moreover, while curcumin’s regenerative potential primarily stems from its ability to reduce inflammation and oxidative stress, asiaticoside demonstrates superior potential for healing chronic wounds. This is due to its involvement in multiple cellular and biochemical processes, such as enhanced collagen synthesis and the activation of specific signaling pathways, which are crucial for effective wound healing and tissue regeneration.

Asiaticoside also exhibits broad-spectrum antibacterial activity, which is crucial for preventing infections in wounds. This activity is enhanced when asiaticoside is incorporated into hydrogels, which provide a sustained release of the compound, thereby maintaining effective antibacterial concentrations over time [28,68]. Hydrogels containing asiaticoside and silver nanoparticles have shown excellent antibacterial properties against common pathogens such as Escherichia coli and Staphylococcus aureus, which are often implicated in wound infections [68].

The antibacterial mechanism of asiaticoside is further supported by its ability to disrupt bacterial cell membranes, as seen in hydrogels that combine asiaticoside with other antibacterial agents such as polylysine [69]. Overall, incorporating asiaticoside into bioscaffolds leverages its natural wound-healing properties while also improving the functionality of the scaffolds, making them more effective for treating chronic and complex wounds.

## 6. Materials and Methods

A comprehensive literature search was performed across Scopus, Medline, and Web of Science (WoS), focusing on publications within the past six years (2019–2024). The search strategy employed a combination of terms related to *Centella asiatica* and its bioactive component, asiaticoside, along with terms associated with scaffolds, biomaterials, and various wound-healing matrices. These were further combined with terms specific to diabetic wounds and ulcers. To maintain the focus and quality of the review, secondary literature and any original articles not published in English were excluded.

## 7. Asiaticoside-Loaded Bioscaffolds: Design and Development

### 7.1. Methods for Incorporating Asiaticoside

Table 2 comprehensively compares various techniques for incorporating asiaticoside into wound-healing scaffolds designed for hyperglycemic conditions. Methods explored include electrospinning, lyophilization, mold casting, and microneedle design. This in-depth analysis provides valuable insights into the effectiveness of each approach for promoting accelerated healing in hyperglycemic wounds. Based on Table 2, the effectiveness or efficacy of the asiaticoside loading techniques can be compared based on several factors including drug release control, biocompatibility, mechanical stability, and scalability. Electrospinning is an effective technique for loading asiaticoside due to its ability to create nanofibrous scaffolds with high surface area, porosity, and ECM-like structure. These properties support controlled drug release and enhance biocompatibility, making electrospun materials specifically advantageous for applications in tissue engineering, particularly for wound healing. However, electrospinning presents challenges in cellular infiltration and mechanical strength, which limit its overall efficacy for providing structural support in tissue engineering. Additionally, the complexity of its fabrication process hampers its scalability for large-scale production.

Freeze drying, especially when used to load asiaticoside in combination with 2-hydroxypropyl-β-cyclodextrin (HPβCD), demonstrates significant efficacy in enhancing the solubility of asiaticoside and ensuring sustained drug release, which is critical for wound healing applications. It excels in preserving scaffold structure, promoting cell infiltration, and maintaining biocompatibility. Despite these advantages, the high cost and energy demands of freeze drying pose limitations to its scalability, particularly in the context of large-scale production.

Casting offers a straightforward and scalable approach for asiaticoside loading, particularly using polymeric nanoparticles. This method allows controlled drug release and is both cost-effective and customizable. However, casting’s limited control over pore structure and interconnectivity affects drug diffusion and cellular infiltration, reducing its overall effectiveness compared to more porous methods such as electrospinning or freeze-drying. Microneedles provide an innovative solution for precise and localized asiaticoside delivery, offering controlled release with minimal pain, making them particularly suitable for targeted treatments. However, they face significant challenges, such as shallow penetration depth, risks of infection, fragility, and high fabrication costs, which limit their broader applicability, especially for deeper tissue treatments.

In summary, freeze drying stands out for its efficacy in providing sustained asiaticoside release and preserving scaffold stability, though scalability remains a concern. Electrospinning is effective in controlled drug release but requires further optimization in terms of structural support and scalability. Casting offers a simple, scalable option but is less effective in ensuring drug release control and cellular interaction. Microneedles excel in targeted, localized delivery, but their use is restricted by penetration depth and production costs. The selection of the most appropriate technique should be guided by the specific therapeutic goals, balancing factors such as efficacy, mechanical properties, and scalability.

### 7.2. Characterization of Asiaticoside-Loaded Bioscaffolds

One study [74] investigated the addition of an excess amount of asiaticoside to various solvents, including methanol, ethanol, acetonitrile, acetone, and water at different pH values (1.2, 4.7, 6.8, and 7.4), while stirring the mixture at 37 °C for 24 h, followed by filtration, and centrifugation to separate undissolved particles. The concentration of asiaticoside in the supernatant was then analyzed using HPLC to quantify its solubility in each solvent. According to the results shown in Table 3, asiaticoside has pH-dependent solubility in water, with higher solubility at higher pH values (up to 270 µg/mL at pH 7.4). Table 4 mentions that asiaticoside is freely soluble in methanol (>20,000 µg/mL) but poorly soluble in acetone, ethyl acetate, and acetonitrile. The addition of surfactants, especially Kolliphor 40 (0.5%), significantly improves asiaticoside’s solubility in water (up to 580 µg/mL). This information is crucial for formulating asiaticoside into effective medicines and skincare products, as solubility impacts its absorption and bioavailability [74]. For asiaticoside loaded in nanoparticles, a Zeta Sizer was used to confirm that the nanoparticles fall within the optimal size range (155 nm to 340 nm) for effective delivery, measure zeta potential to evaluate stability, and calculate the polydispersity index to asses for uniform particle distribution, ensuring predictable drug behavior [78].

To examine the scaffold’s structure, Transmission Electron Microscopy (TEM) was used to reveal its internal structure and composition [75]. Surface morphology was assessed using a combination of Atomic Force Microscopy (AFM), Field Emission Scanning Electron Microscopy (FESEM), and Scanning Electron Microscopy (SEM) [61,74,75]. These techniques collectively provide a comprehensive understanding of the bioscaffold’s properties, which is essential for evaluating its potential for controlled drug delivery applications. The cryo-SEM analysis of a bioscaffold involved preparing the hydrogel, rapidly freezing it to preserve its structure, imaging the frozen samples under vacuum for high-resolution results, and analyzing the images generated. This method was specifically employed to visualize the hydrogel’s porous network structure, which is beneficial for the transport of oxygen and nutrients during wound healing, and to assess the overall morphology of the hydrogel [79].

For tissue engineering applications such as wound healing, it is especially important to measure the porosity of scaffolds. Scanning Electron Microscopy (SEM), when combined with the liquid displacement method, offers a thorough way of precisely determining scaffold porosity [75]. The liquid displacement method measures porosity by quantifying the volume of liquid that penetrates the pores, whereas SEM provides intricate visual details regarding the morphology and architecture of those pores [80]. As listed in Table 5, a variety of characterization techniques were used to evaluate the physicochemical characteristics and drug delivery capacity of asiaticoside-loaded bioscaffolds

Table 5 mentions the methods used to characterize the properties of the bioscaffold. According to [74], fluid uptake studies were performed by immersing the hydrogels in distilled water at 37 °C. The weight of each hydrogel sample was recorded at regular intervals until it reached a state of equilibrium swelling, indicated by a constant weight. The importance of a stable swelling ratio for scaffolds is its impact on the mechanical properties, porosity, and permeability of scaffolds, which are essential for cell attachment and nutrient exchange in tissue engineering. It also enables controlled drug release from delivery systems, and ensuring consistent drug release rates [80]. Other methods of assessing a scaffold’s swelling ratio can be performed by gravimetric methods or immersion in PBS [61]. PBS is preferred for the swelling ratio of hydrogels, as it simulates physiological conditions (pH 7.4), facilitating accurate assessment of fluid absorption, essential for wound dressings. PBS also minimizes changes in ionic strength and pH, ensuring consistent results. The gravimetric method offers a quantitative evaluation of the material’s effectiveness regarding its ability to absorb fluids and its degradation behavior [61].

Moisture transmission across scaffolds was evaluated using a Water Vapor Transmission Rate (WVTR) assay. It can be performed by mounting the scaffolds on a centrifuge tube containing deionized water, sealed to prevent leakage, and incubating the assembly at 37 °C. After 24 h, the weight loss is measured [81]. The WVTR results of bioscaffolds emphasize the importance of maintaining an optimal moisture level for wound healing, showing that both extremely high and low WVTRs can negatively affect the healing process [82].

Additionally, the table lists methods to determine gelation time/polymerization time (inverted tube method) and tissue adhesion capacity (adhesion test). These techniques collectively provide a comprehensive understanding of scaffolds’ properties relevant to their application in tissue engineering and drug delivery.

To determine the amount of asiaticoside loaded into the bioscaffolds, an entrapment efficiency (EE%) test was performed. The EE% was determined using a destructive sampling method. A specific volume of the nano-formulation was mixed with a methanol/acetone solvent, followed by sonication and centrifugation. The supernatant was analyzed using HPLC to quantify the drug, and the EE% was calculated based on the amount of drug entrapped compared to the total amount used for formulation [74]. Entrapment efficiency is a crucial validation step for optimized scaffold formulations, measuring how well the formulation meets drug loading and release criteria for effectiveness in biomedical applications [83].

Globally, researchers used spectroscopy to confirm the successful combination of asiaticoside with other materials in a new formulation. By analyzing the absorption spectra, they identified distinct peaks characteristic of asiaticoside and the combined materials, confirming the creation of a new material with unique properties. The presence of these different peaks provides evidence of successful integration, highlighting the potential of this method for developing and characterizing asiaticoside-based drug delivery systems [75].

Additionally, researchers utilized fluorescent imaging to assess both the release kinetics of the active substance and the microneedle insertion depth. Asiaticoside-loaded microneedles containing Indocyanine Green (ICG) fluorescent dye were inserted into pig skin, allowing real-time visualization of dye distribution and confirmation of successful drug delivery [75]. This real-time imaging capability is crucial for guiding treatment and evaluating therapeutic efficacy, as the fluorescence emitted by ICG provides immediate feedback on drug localization within the tissue [84]. Other methods include dialysis bag diffusion monitored with HPLC and immersion in phosphate-buffered saline (PBS) followed by UV spectroscopy analysis [61,74,75,79]. To evaluate asiaticoside release, researchers used a dialysis bag method where asiaticoside-loaded scaffolds were enclosed within a semi-permeable membrane and immersed in a buffer solution. Samples were collected over time and analyzed using HPLC to measure the amount of asiaticoside released, providing insights into its release kinetics [74].

The mechanical properties of the scaffolds were thoroughly evaluated using various techniques. The tensile strength can be determined using a Zwick Roell Z005 tensile testing machine [61]. Scaffolds need sufficient tensile strength to withstand physiological loads and prevent collapse, which can impair healing by hindering tissue formation and delaying recovery [85]. A rheometer was employed to assess the viscosity and dynamic elastic modulus, while a universal testing machine was used to measure the compression modulus [75,79]. Rheological tests are crucial for evaluating wound healing scaffolds. These tests not only characterize the scaffold’s viscoelastic behavior, ensuring that it mimics the dynamic mechanical environment of natural tissue, but also assess its stress relaxation behavior, which is critical for long-term support of tissue healing and integration. This comprehensive evaluation ensures the scaffold can effectively function within the body [86]. As for compression testing of wound-healing scaffolds, it aids in selecting appropriate materials and designs for scaffolds based on their mechanical performance. This ensures that the final product meets the requirements for clinical applications [87]. These comprehensive measurements provide valuable insights into a scaffold’s mechanical behavior and suitability for its intended application.

The biodegradability of scaffolds can be tested by immersing the scaffolds in type I collagenase enzyme and PBS mixed with lysozyme [61,75,81]. Collagen type I is chosen for biodegradation testing due to its pivotal role in tissue structure and its biocompatibility. As a primary component of the extracellular matrix, it closely resembles the body’s natural environment. Moreover, its regulated degradation by metalloproteinase enzymes ensures a biocompatible and controlled breakdown process, essential for wound-healing applications [88]. A mixture of PBS and lysozyme can be used to simulate a physiological environment and enzymatic degradation, providing a controlled and relevant method for comparing the biodegradability of different scaffolds [89].

## 8. Preclinical and Clinical Evaluation

### 8.1. In Vitro Studies

Table 6 presents a summary of in vitro studies investigating the efficacy of asiaticoside-loaded bioscaffolds in promoting wound healing. A range of scaffold materials, such as hydrogels, 3D-printed scaffolds, and nanofibrous membranes, were evaluated; each of these materials included asiaticoside in its scaffold formulation. Cell viability assays, cell migration assays, and specialty testing such as collagen synthesis assays and microbiological penetration tests were among the in vitro studies carried out. All of the findings point to the asiaticoside-loaded bioscaffolds’ potential use in wound healing by demonstrating their ability to improve collagen synthesis, boost cell viability, enhance cell migration, and show antibacterial qualities.

### 8.2. In Vivo Studies

An overview of the many in vivo tests used to evaluate the effectiveness of various wound healing treatments is provided in Table 7. These tests include histological examinations, evaluations of wound healing in diabetic mice, and evaluations of biocompatibility. Numerous techniques were applied, such as flow cytometry, immunofluorescence, and histological labeling. The outcomes regularly show better tissue regeneration, higher collagen deposition, wound healing, and efficient inflammatory response management. The treatments mentioned specifically were hydrogels and scaffolds loaded with asiaticoside. The PNP group that was loaded with asiaticoside had a noteworthy rise in collagen levels. Some research utilized biochemical assays to assess healing at a molecular level, even though the majority of investigations concentrated on histological and cellular alterations.

### 8.3. Synergistic Effects with Other Biomaterials

The synergistic effects observed when combining asiaticoside with other biomaterials have garnered significant attention in the field of wound-healing research. This review delves into the mechanisms behind these interactions, focusing on how asiaticoside, when combined with biomaterials, can significantly accelerate and improve wound-healing outcomes, as summarized in Table 8.

### 8.4. Preclinical Trials

Several preclinical trials have examined the efficacy of asiaticoside-loaded scaffolds for wound healing in diabetic or hyperglycemic conditions. One such study involved nanofiber scaffolds loaded with asiaticoside, tested on diabetic rat models. The rats were divided into four groups: toxic control (TC), normal/placebo control (NC), non-crosslinked nanofiber scaffold (F1), and cross-linked nanofiber scaffold (F2). Wound areas were assessed on days 0, 3, 6, 9, and 14. The F1 and F2 groups showed enhanced wound healing rates, with the F2 group demonstrating the most significant improvement starting from day 3 and continuing through day 14. In contrast, the TC and NC groups only showed minor improvements after day 6 or day 9. Histopathological analysis confirmed these results, showing improved tissue regeneration, reduced inflammation, and better collagen synthesis in the F1 and F2 groups compared to controls. Tissue samples were collected at days 0, 7, and 14; stained with hematoxylin and eosin (H&E); and evaluated for indicators of wound healing, including epithelial regeneration, cellular composition, collagen synthesis, and inflammation. The treated groups exhibited successful re-epithelialization, increased fibroblasts, decreased inflammatory cells, and organized collagen synthesis. By day 14, the wounds in the treated groups reached the maturation and remodeling stages, confirming the therapeutic potential of asiaticoside-loaded nanofibrous scaffolds for diabetic wound treatment [61].

Another example would be preclinical trials conducted on asiaticoside polymeric nanoparticles (AST PNP) that were loaded into a gelatin-based hydrogel scaffold to evaluate the therapeutic efficacy on diabetic wound healing. In vitro studies on mouse dermal fibroblasts (L929) assessed the cytocompatibility of AST PNP through the MTT assay, confirming its non-toxic nature and support for cell viability. Additionally, AST PNP increased the synthesis of collagen, particularly collagen type I, essential for the structural integrity of healing tissue, and provided protective effects against oxidative stress. These findings highlighted AST PNP’s ability to improve cellular processes crucial for effective wound healing, setting a strong foundation for its in vivo evaluation.

In vivo studies in diabetic rats demonstrated significant wound healing improvement with AST PNP gel treatment, achieving complete wound closure by day 21. Histopathological analysis showed a thicker epidermal layer and more organized tissue structure in the AST PNP-treated group, along with denser and more organized collagen fibers. Collagen quantification indicated a higher content of collagen type I (COL-1) in AST PNP-treated wounds. Immunohistochemical analysis revealed elevated α-SMA expression, indicating enhanced differentiation of fibroblasts into myofibroblasts and improved wound contraction. The gelatin scaffold provided sustained release of asiaticoside over 24 h, maintaining therapeutic levels at the wound site and promoting continuous healing stimulation. These comprehensive findings from in vitro and in vivo studies highlighted the efficacy of AST PNP in promoting wound healing, setting the stage for potential clinical applications [74].

### 8.5. Clinical Trials

Despite the promising preclinical discoveries shown in this review, a significant scarcity of clinical trials studying asiaticoside-loaded bioscaffolds for wound healing in humans was observed. This lack of clinical data highlights the necessity of future research to translate these promising preclinical results into human applications, eventually determining the safety and efficacy of this approach for patients with diabetic foot ulcers. Clinical trials encounter numerous challenges, including funding restrictions, reproducibility concerns, disease intricacy, regulatory limitations, methodological variability, lack of interdisciplinary collaboration, and high failure rates. Successfully designing new therapies demands better strategies and collaboration to overcome these hurdles in clinical trials [90].

## 9. Analysis of Differences in Experimental Results

The experimental results across the bioscaffolds reveal distinct approaches to diabetic wound healing, highlighting the variations in their efficacy, mechanisms, and therapeutic outcomes, and a comparative analysis of the results can be found in Table 9. The dual-crosslinked hydrogel demonstrated significant antibacterial activity and fibroblast proliferation in vitro, showcasing its potential to combat infections and promote cellular growth. In vivo, it achieved substantial improvements in wound closure rates compared to single-crosslinked formulations, emphasizing its innovativeness in providing sustained release of therapeutic agents. However, its limited ability to adapt to complex wound shapes presents a constraint.

The asiaticoside nanoparticles showed an emphasis on sustained drug release and enhanced fibroblast migration, which were linked to collagen synthesis. In vivo results confirmed faster wound closure and superior collagen deposition in diabetic rats, reflecting a strong focus on ECM reconstruction and angiogenesis. While the nanoparticles offered improved bioavailability, the scalability of nanoparticle fabrication could hinder broader application.

The printable hydrogel introduced immune modulation as a key mechanism, orchestrating macrophage responses to resolve chronic inflammation and accelerate wound healing. Its rapid closure rates in vivo, particularly in diabetic chronic wound models, demonstrated its ability to adapt to wound shapes effectively. However, its mechanical strength was inferior to that of nanofibers or microneedles, making it less robust in challenging wound environments.

The nanofibrous scaffold, with its unique combination of low cytotoxicity, antimicrobial efficacy, and sustained release, demonstrated enhanced tensile strength and vascularization in diabetic rats. The in vivo studies showed excellent outcomes in restoring skin architecture and reducing inflammation, though the slower degradation rate could limit the scaffold’s adaptability in dynamic healing processes.

The MXene microneedle stood out for its innovative design, combining mechanical testing with fibroblast proliferation and angiogenesis studies. It achieved accelerated healing with reduced fibrosis in diabetic mice, facilitated by its ability to penetrate the cuticle and deliver drugs subcutaneously with prolonged release. Despite its advanced results, the cost of MXene synthesis and integration presents a challenge for scalability.

The differences in experimental results reflect the specialized focus of each bioscaffold. Hydrogels excel in creating moist environments and immune modulation, while nanoparticles and microneedles focus on advanced drug delivery mechanisms. Nanofibrous scaffolds balance mechanical properties and tissue regeneration. The choice of bioscaffold depends on the wound complexity, therapeutic requirements, and cost considerations, with each offering distinct advantages tailored to specific clinical needs

## 10. Discussion

### 10.1. Mechanisms of Asiaticoside in DFU Healing

Table 10 summarizes the key mechanisms of asiaticoside in promoting diabetic wound healing, as identified from various research studies. Asiaticoside, a bioactive compound derived from *Centella asiatica*, exhibits multifaceted effects, including promoting collagen synthesis, enhancing angiogenesis, reducing inflammation, and stimulating cell proliferation and migration. These properties collectively contribute to its potential as a therapeutic agent for accelerated wound healing in diabetic patients.

### 10.2. Limitations and Corresponding Solutions for Asiaticoside-Loaded Bioscaffolds

While asiaticoside-loaded bioscaffolds hold immense promise for tissue regeneration, it is crucial to recognize and address their limitations. These limitations primarily originate from the properties of asiaticoside and the challenges associated with its incorporation into a bioscaffold.

One major limitation is the complexity and cost of obtaining purified asiaticoside from *Centella asiatica*. Extraction processes are often inefficient, delaying large-scale production and potentially influencing bioavailability [81]. A favorable option is to utilize whole plant extracts, which are more readily available and inexpensive [91]. This method also allows a synergistic effect of various bioactive compounds in the extract, potentially enhancing therapeutic outcomes. However, standardization of plant extracts is crucial to ensure consistency and efficacy. Techniques including High-Performance Liquid Chromatography (HPLC); Gas Chromatography (GC); Mass Spectrometry (MS); and spectroscopic methods such as Nuclear Magnetic Resonance (NMR) spectroscopy, Infrared (IR) spectroscopy, and Near-Infrared (NIR) spectroscopy can address this concern [92].

Asiaticoside’s poor solubility, low bioavailability, and limited cell penetration pose significant challenges to its effective delivery to the wound site. Just incorporating asiaticoside into a bioscaffold may not ensure its therapeutic efficacy [61]. To overcome this, advanced drug delivery systems and novel biomaterials are necessary.

Encapsulation techniques are used to protect plant bioactive ingredients from degradation, enhance their solubility and bioavailability, and achieve controlled release. These techniques involve combining the bioactive components into various delivery systems, such as liposomes, nanoparticles, and nanoemulsions. This process offers several benefits, including improved stability during processing and storage, enhanced bioavailability, controlled release for targeted delivery, and incorporation into matrices without affecting quality characteristics. The selection of the delivery system and encapsulation method depends on the specific bioactive elements and their intended applications [93].

## 11. Future Directions

While promising, asiaticoside-loaded bioscaffolds still face challenges that must be addressed to maximize their therapeutic potential. Huang et al. [94], suggests that future research on asiaticoside-loaded bioscaffolds for wound healing will focus on enhancing their strength and compatibility with the body, as it is one of the challenges posed to current research. This can be achieved by investigating more biomaterials, such as hybrid composites or bioinspired polymers, that combine the advantages of natural and synthetic scaffolds. Further analysis of their degradation in the body and effects on the wound should also be conducted. This could help to improve the biocompatibility and longevity of the bioscaffolds. Furthermore, advanced fabrication techniques such as 3D bioprinting, electrospinning, and freeze drying could be used to refine scaffold architecture at the microscale and nanoscale, improving mechanical strength and mimicking the extracellular matrix for better cell interaction. Wang et al. [75], proposes investigating the synergistic effects of combining asiaticoside with other treatments such as growth factors to improve wound healing further. Additionally, Naseem et al. [64], highlights the need for ongoing research to optimize the formulation of asiaticoside-loaded bioscaffolds for improved therapeutic results. Furthermore, optimizing the formulation of asiaticoside-loaded bioscaffolds is crucial for maximizing their therapeutic potential [61]. This includes refining their composition, fabrication procedures, and drug delivery abilities, such as improving drug loading and release profiles, and improving mechanical properties. Achieving this may involve exploring different biomaterial ratios or using asiaticoside in combination with other bioactive agents.

The future direction of asiaticoside-loaded bioscaffolds lies in personalized medicine, as emphasized by [61,75]. This involves tailoring the scaffold and asiaticoside delivery to personal patient needs. Factors to assess include the precise type of diabetes, unique wound characteristics, and personal responses to treatment. It is important for this research strategy to be used, as different bodies will react differently to the bioscaffold in promoting wound healing process. This personalized approach aims to optimize wound healing by providing more precise and effective therapy that would help in combating any challenges faced during the process of creating the formulation and incorporating asiaticoside into different bioscaffolds.

Asiaticoside-loaded bioscaffolds have demonstrated promising results in preclinical studies, as indicated by multiple researchers [61,75,81]. This growth involves rigorous clinical trials to assess the safety and efficacy of these innovative wound treatments in humans with diabetic foot ulcers. These trials are not only confirmation of preclinical findings but also a crucial step towards personalized medicine. As proposed by Anand et al. [61], treatment protocols must be optimized based on individual patient profiles, including the severity of their diabetes, the specific features of their wounds, and their responses to therapy.

Additionally, Wang et al. [72] & Wang et al. [81], highlight the significance of translating these research findings into real-world clinical settings, that is, proving the effectiveness of asiaticoside-loaded bioscaffolds in treating diabetic wounds in diverse patient populations and under typical healthcare conditions. Eventually, successful clinical trials will pave the way for widespread application of this promising technology, giving hope for improved healing and quality of life for individuals suffering from diabetic foot ulcers.

The success of asiaticoside-loaded bioscaffolds hinges not only on their clinical effectiveness but also on their manufacturability. As suggested by Anand et al. [61], exploring scalable manufacturing processes is crucial for both commercial viability and overall clinical adoption. Overall, the future research strategy to solve any bottlenecks found in the development of asiaticoside-loaded bioscaffolds relies on overcoming these challenges through a combination of material innovation, advanced fabrication techniques, personalized approaches, and successful clinical translation.

## 12. Conclusions

This review has studied the potential of bioscaffolds loaded with asiaticoside to change the treatment of hyperglycemic wounds. Derived from *Centella asiatica*, asiaticoside displays a variety of characteristics that effectively tackle the complicated issues related to diabetic wounds, such as decreased angiogenesis, prolonged inflammation, and delayed tissue regeneration. Researchers have successfully explored asiaticoside’s medicinal effects by incorporating it into different bioscaffold designs, thereby using the specific advantages of each scaffold type.

The adaptability of this method is shown by the wide range of asiaticoside-loaded bioscaffolds mentioned, including hydrogels, microneedle arrays, and nanofibrous meshes. Studies conducted both in vivo and in vitro have regularly shown the successfulness of these scaffolds at increasing collagen synthesis, promoting angiogenesis, lowering inflammation, and increasing wound closure rates. The synergistic effects of asiaticoside in combination with other biomaterials including gelatin, silk fibroin, PVA, and others emphasize the potential of developing even more effective treatment approaches.

Even if preclinical research has shown positive outcomes, these findings must be applied in clinical settings. Subsequent investigations need to concentrate on carrying out thorough clinical trials to assess the safety and efficacy of asiaticoside-loaded bioscaffolds in individuals with DFUs. General applications will also depend on manufacturing process optimization to ensure scalability and affordability.

Asiaticoside-loaded bioscaffolds, in summary, comprise a significant development in the field of wound healing. Their ability to tackle the complex issues associated with hyperglycemic wounds, in addition to delivering tailored treatment options, makes them a potentially effective therapeutic approach for enhancing the quality of life of those with diabetes.

## Figures and Tables

**Table 1:** Wagner–Meggitt grading system for diabetic foot ulcers.

Grade	Description
0	Foot symptoms such as pain only
1	Superficial ulcers involving skin and subcutaneous tissue
2	Deep ulcers involving ligaments, muscles, tendons, etc.
3	Ulcer with bone involvement
4	Forefoot gangrene
5	Full-foot gangrene

Source: [18].

**Table 2:** Comparison of techniques for loading asiaticoside into scaffolds for hyperglycemic wound healing.

Reference No.	Loading Technique	Description	Advantages	Disadvantages
[61,70]	Electrospinning	Polyvinyl alcohol/sodium alginate-silk fibroin/based nanofibrous scaffolds loaded with asiaticoside.	Electrospinning creates nanofibers with high surface area, porosity, and versatility for material combinations, allowing controlled drug release and biocompatibility, all of which is beneficial for wound healing.	Poor cellular infiltration, inadequate mechanical strength for the scaffolds, limited production scalability, potential cytotoxicity, and complex fabrication parameters.
[71,72]	Freeze drying	Gelatin scaffolds loaded with asiaticoside/2-hydroxypropyl-β-cyclodextrin complex were fabricated using freeze drying.	Preserving scaffold structure, controlling porosity; improving stability, shelf life, and drug release; maintaining biocompatibility; creating lightweight and easy-to-handle scaffolds; and facilitating cell infiltration.	The process is both costly and energy-intensive, hindering large-scale scaffold production and sustainability.
[73,74]	Casting	The gelatin solution containing the asiaticoside polymeric nanoparticles is cast into molds, allowed to gel, and crosslinked with glutaraldehyde.	Simple, versatile technique that allows controlled drug loading and crosslinking. It is also scalable and customizable.	Limited control of pore structure and interconnectivity.
[75,76,77]	Microneedles	Two hydrogels were formulated: (1) MXene–AS hydrogel: Ti_3_C_2_ MXenes were mixed with asiaticoside solution. (2) γ-PGA hydrogel: γ-PGA was dissolved in water and combined with the MXene–AS mixture. These hydrogels were used to create microneedles. Microneedle dressings were made from polysulfobetaine methacrylate (PSBMA), photothermal hair particles (HMPs), zinc oxide (ZnO NPs), and asiaticoside.	Enhanced drug delivery, minimized pain, good moisture retention, controlled drug release, improved biocompatibility, and versatility in formulation.	Insufficient penetration depth, fragility, infection risks, pain during application, high costs, and biocompatibility issues with non-biodegradable tips.

**Table 3:** Solubility of asiaticoside at different pH values.

pH	Solubility (µg/mL)
1.2	230
4.7	250
6.8	260
7.4	270

**Table 4:** Solubility of asiaticoside in various solvents and surfactants.

Solvent	Solubility (µg/mL)
Methanol	20,000
Acetone	150
Ethyl acetate	20
Acetonitrile	>10
Kolliphor 20 (0.5%)	580
PEG 400 (2%)	210
Propylene glycol (5%)	220
Kolliphor 40 (0.5%)	380
Tween 80 (0.5%)	300
PVA (0.5%)	100

**Table 5:** Asiaticoside-loaded bioscaffolds: characterization and release profiles.

Reference No.	Property	Characterization Method	Results/Outcomes
[74]	Scaffold material	Solubility of asiaticoside in various solvents and buffer solutions	Solubility profile of asiaticoside
		Zeta Sizer	Physicochemical characterization of asiaticoside-loaded polymeric nanoparticles
[75]	Scaffold structure	TEM	Internal structure and composition of the scaffold
[61,74,75]		AFM, FESEM, and SEM	Surface morphology
[78]		Cryo-SEM	Surface topography of the scaffold
[81]	Scaffold porosity	SEM	Microstructure of the scaffold
		Liquid displacement method	Structural integrity of the scaffold
[74]	Scaffold properties	Fluid uptake study	Fluid retention in the scaffold
[74]		Rate of water evaporation	
[61]		Gravimetric method for swelling ratio	
[81]		Immersion in PBS for swelling ratio	
[81]		Immersion in distilled water	
[74,81]		WVTR assay	Moisture transmission across the dressing
[79]		Inverted tube method	Gelation time/polymerization time
[79]		Adhesion test	Tissue adhesion capacity
[74]	Asiaticoside loading	Entrapment efficiency (%)	Loading capacity of the asiaticoside-loaded polymeric nanoparticles
[75]	Drug release	UV-Vis spectroscopy	Chemical interaction and stability of the scaffold formulation—successful asiaticoside or drug incorporation into the scaffold
[75]		Fluorescent assay	Release kinetics of asiaticoside
[74,79,81]		Dialysis bag method—observe using HPLC	
[61]		Immersion in PBS and observation by UV spectroscopy	
[61]	Mechanical properties	Tensile testing machine (e.g., Zwick Roell Z005 model)	Tensile strength of the scaffold
[79,81]		Rheometer	Viscosity and dynamic elastic modulus of the scaffold
[79,81]		Universal testing machine	Compression modulus of the scaffold
[75,81]	Biodegradability	Type I collagenase enzyme	Biodegradation of the scaffold
[61]		PBS + lysozymes	

**Table 6:** In vitro evaluation of asiaticoside-loaded bioscaffolds for wound healing.

Reference No.	Scaffold Properties	In Vitro Test Performed	Results
[75]	Cell viability	Live/dead cell double staining with confocal microscopy	No cytotoxic effects on HUVECs after 1 and 3 days
[74]		MTT assay with L929 mouse dermal fibroblasts	Asiaticoside-loaded PNP showed good cytocompatibility no adverse effects on cell viability
[61]		MTT assay with HaCaT cells	Asiaticoside-loaded nanofibers showed less cytotoxicity and good biocompatibility
[81]		MTT assay with fibroblasts (NIH 3T3)	CA@3D scaffold extracts significantly enhanced fibroblast viability compared to the control
[79]	Cytocompatibility	CCK-8 assay with rabbit red blood cells, then live/dead staining	Comparable cell proliferation in hydrogel groups Live/dead staining showed good cell viability
[75]	Migration assay	Scratch wound assay with fibroblasts	MN–MXene–AS extract significantly enhanced fibroblast migration over 24 h
[74]		Scratch assay with L929 cells	Asiaticoside-loaded PNP enhanced wound closure, suggesting improved wound-healing properties
[61]		Scratch assay with HaCaT cells	Significant cell migration and re-epithelialization promoted by the treated nanofibers
[81]		Scratch assay with fibroblasts	CA@3D scaffold extract significantly accelerated scratch closure, indicating enhanced fibroblast migration
[74]	Cellular uptake	Fluorescence microscopy with coumarin-6-loaded PNP	Effective cellular internalization in L929 fibroblasts
[61]	Antibacterial activity	Time-kill assay against *P. aeruginosa* and *S. aureus*	Significant reduction in bacterial counts over time
[79]		Minimum inhibitory concentration (MIC) determination against *S. aureus* and *E. coli*, optical density (OD) measurements to assess bacterial growth, live/dead staining	Hydrogel showed significant antibacterial activity, with membrane disruption in treated bacteria
[61]	Microbial penetration	Nutrient broth penetration test	Resisted microbial penetration, indicating potential for infection prevention
[81]	Collagen synthesis	Collagen quantification assay (likely colorimetric)	CA@3D scaffold extract significantly increased collagen synthesis compared to the control
[81]	Anti-inflammatory test	Measurement of pro-inflammatory cytokine (TNF-α, IL-6) levels after LPS stimulation	CA@3D scaffold extract significantly reduced pro-inflammatory cytokine levels
[79]	Hemocompatibility	Hemolysis rate calculation using rabbit red blood cells	The hemolysis rate was <5%, indicating good hemocompatibility

**Table 7:** In vivo evaluation of asiaticoside-loaded bioscaffolds for wound healing.

Reference No.	Scaffold Properties	In Vivo Test Performed	Results
[74,75]	Wound healing	In vivo wound healing evaluation in diabetic mice	Faster wound closure, enhanced collagen synthesis, and better tissue regeneration compared to the control and other treatment groups.
[61,79]			Enhanced wound closure rate, histological analysis, and tissue regeneration.
[81]			Significantly faster healing than controls, with complete closure by day 10.
[74]	Histological analysis	Histopathological studies of healed tissues in diabetic rats	The asiaticoside-loaded scaffold-treated group showed a thicker epidermal layer, denser collagen deposition, and well-organized collagen fibers, indicating enhanced extracellular matrix (ECM) formation.
[61]		Histological analysis of wound tissue at various time points	Improved re-epithelialization and increased collagen deposition in wounds treated with nanofibrous scaffolds compared to controls, indicating enhanced tissue regeneration and healing.
[81]		Wound tissues analyzed using H&E and Masson staining to assess tissue morphology, epithelial thickness, collagen, and hair follicles	Increased epithelial thickness, collagen, and hair follicles in treated wounds.
[81]		Tissue sections stained for CD31 (angiogenesis marker) and analyzed via confocal microscopy	Higher CD31 expression in treated wounds, indicating enhanced angiogenesis.
[81]		Macrophage polarization analysis: macrophage types (M1/M2) in wound tissues analyzed by flow cytometry on days 3, 7, and 10	Shift from M1 to M2 macrophages in treated wounds, indicating effective inflammation modulation.
[74]	Biochemical assays	Biochemical assays of healed tissues in diabetic rats	The asiaticoside-loaded PNP group showed a significant increase in collagen levels and upregulation of COL-1 protein levels, indicating enhanced collagen biosynthesis and ECM remodeling.
[79]	Biocompatibility assessment	Hydrogels were implanted under the skin of rats; tissues collected and analyzed with H&E staining	Hydrogel showed biocompatibility (specific results on tissue response not detailed).

**Table 8:** Synergistic effects of asiaticoside combined with various biomaterials in diabetic wound healing.

Reference No.	Biomaterial Combined with Asiaticoside	Mechanism of Action	Key Findings
[75]	Poly-γ-glutamic acid (γ-PGA)	Primary matrix for microneedles Biocompatible and biodegradable Maintains a moist wound environment Facilitates controlled release of asiaticoside	Controlled release of asiaticoside within 20 min Improved cell viability and migration in vitro
[75]	MXenes (Ti_2_C_3_)	Enhances mechanical strength of microneedles Improves rigidity and penetration capability Excellent drug-loading capabilities and biocompatibility	Enhanced mechanical strength for effective skin penetration
[74,81]	Gelatin	Natural polymer derived from collagen Biocompatible, biodegradable Excellent cell adhesion properties Provides scaffold for cell attachment and migration	Enhanced wound closure (complete closure by day 21) Improved collagen deposition (denser and well-organized collagen fibers) Thicker epidermal layer in regenerated skin tissue
[74,81]	Polymeric nanoparticles (polycaprolactone (PCL))	Loaded with asiaticoside Provides sustained release of asiaticoside	Sustained release of asiaticoside for prolonged therapeutic effect Enhanced collagen synthesis and ECM remodeling Increased collagen levels and expression of COL-1 and α-SMA
[61,81]	Silk fibroin (SF)	Excellent biocompatibility, mechanical strength Supports cell attachment and proliferation Promotes tissue regeneration Favorable properties for drug delivery	Enhanced mechanical properties of the scaffold Provided a suitable environment for cell growth and migration
[61,81]	Polyvinyl alcohol (PVA)	Hydrophilic polymer for moisture retention Contributes to scaffold flexibility and biodegradability	Maintained a moist wound environment Facilitated faster healing and reduced scab formation
[61,81]	Sodium alginate (SA)	Excellent water-absorbing properties Promotes cell migration Antimicrobial properties Forms a stable hydrogel network when crosslinked	Aids in maintaining moisture Provides a barrier against microbial invasion Provides a scaffold for cell attachment and growth
[79]	*ϵ*-Poly-L-lysine (*ϵ*-PLL)	Provides structural integrity to the hydrogel Exhibits antibacterial properties against Gram-positive and Gram-negative bacteria	Reduces the risk of infection in wounds
	4-armed poly(ethylene glycol) (4aPEG)	Serves as a crosslinker in the hydrogel Contributes to the hydrogel’s mechanical properties	Enhances the stability and durability of the hydrogel

**Table 9:** Analysis of differences in experimental results.

Bioscaffolds	Dual-Crosslinked Hydrogel [79]	Asiaticoside Nanoparticles [74]	Printable Hydrogel [81]	Nanofibrous Scaffold [61]	MXene Microneedle [75]
In vitro studies	Antibacterial activity, fibroblast proliferation	Sustained drug release, fibroblast migration, and collagen synthesis	Immune modulation through macrophage regulation	Cell migration, low cytotoxicity, antimicrobial efficacy	Mechanical testing, fibroblast proliferation, angiogenesis
In vivo model	Diabetic mice	Diabetic rats	Diabetic chronic wounds in mice	Diabetic rats	Diabetic mice
Wound closure rates	Significant improvement compared to single-crosslinked formulations	Faster closure and enhanced collagen synthesis	Rapid closure with immune pathway activation	Enhanced closure with tensile strength and vascularization	Accelerated healing with reduced fibrosis
Drug release mechanism	Sustained release via hydrogel matrix	Prolonged release from polymeric nanoparticles	Controlled release through 3D printed scaffold	Extended release via nanofiber encapsulation	Slow release enabled by MXene nanosheets
Key mechanisms	Antibacterial and ECM mimicry	Collagen biosynthesis and angiogenesis	Macrophage-mediated immune modulation	Reduced inflammation and enhanced vascularization	Epithelization and controlled angiogenesis
Strengths	Antibacterial properties and moist wound environment	Sustained delivery with superior bioavailability	Cost-effective and adaptable for wound shape	High drug loading and mechanical stability	Penetrates cuticle for efficient subcutaneous drug delivery
Limitations	Limited customization for complex wounds	Dependence on nanoparticle fabrication efficiency	Lower mechanical strength than nanofibers or microneedles	Slower degradation compared to hydrogels	Cost of MXene synthesis and integration

**Table 10:** Mechanisms of action of asiaticoside on diabetic wounds.

Reference No.	Action	Description
[74,75]	Promotes cell proliferation	Promotes fibroblast proliferation and migration Facilitates their transition to myofibroblasts for enhanced wound contraction
[61,75,79,81]	Regulates angiogenesis	Influences angiogenesis in a biphasic manner, promoting it during inflammation and modulating it during remodeling
[74]		May enhance VEGF expression, promoting angiogenesis and improving blood supply for nutrient and oxygen delivery to the wound area
[75]	Reduces inflammation	NF-κB p65 translocation, a key transcription factor in inflammatory responses
[61,79]		Inhibits pro-inflammatory signaling pathways and down-regulates pro-inflammatory factors, reducing the inflammatory response of macrophages
[79]		Decreases the expression of pro-inflammatory cytokines such as TNF-α, promoting a conducive healing environment
[79]		Increases the expression of anti-inflammatory markers such as IL-10
[75]	Downregulates pro-inflammatory cytokines	Downregulates TNF-α expression, reducing vascular permeability and chronic inflammation
[74]		Has anti-inflammatory properties, creating a more favorable environment for diabetic wound healing
[75]	Modulates extracellular matrix (ECM) components	Asiaticoside alters expression of genes encoding products such as collagen (COL1A2, COL3A1) and MMPs, crucial for structural integrity and remodeling of the ECM
[74]		Restores ECM tissue architecture
[75]	Dynamically regulates gene expression	Regulates wound-healing genes, including LOX, LOXL3, TIMP1, and CHI3L2, influencing cell behavior and ECM remodeling
[74]	Promotes collagen synthesis	Enhances the production of collagen type I (COL-1), improving the structural integrity and strength of regenerated tissue
[61,81]		Stimulates fibroblast proliferation, essential for ECM formation and tissue repair
[74]	Reduces oxidative stress	Has antioxidant properties that mitigate oxidative stress to prevent tissue damage
[74]	Enhances cellular uptake	Formulating asiaticoside into polymeric nanoparticles improves its solubility, permeability, and cellular uptake at the wound site
[61,79,81]	Stimulates cell migration	Facilitates keratinocyte and fibroblast migration to the wound site for re-epithelialization and tissue regeneration
[61]	Exerts anti-microbial effects	Prevents infections in diabetic wounds
[61]	Regulates protease activity	Prevents the degradation of essential ECM proteins
[81]	Regulates macrophage phenotypes	Promotes a shift in macrophage populations from the pro-inflammatory (M1) phenotype to the anti-inflammatory (M2) phenotype, facilitating the resolution of inflammation
[79]	Reduces scar formation	Causes a reduction in scar formation for better aesthetic and functional outcomes

## Data Availability

The data presented in this study are available on request from the corresponding author.

## References

[1] Singh S., Young A., McNaught C. (2017). The physiology of wound healing. Surgery.

[2] Krzyszczyk P., Schloss R., Palmer A., Berthiaume F. (2018). The Role of Macrophages in Acute and Chronic Wound Healing and Interventions to Promote Pro-wound Healing Phenotypes. Front. Physiol..

[3] Rajendran N.K., Kumar S.S.D., Houreld N.N., Abrahamse H. (2018). A review on nanoparticle-based treatment for wound healing. J. Drug Deliv. Sci. Technol..

[4] Almadani Y.H., Vorstenbosch J., Davison P., Murphy A. (2021). Wound Healing: A Comprehensive Review. Semin. Plast. Surg..

[5] Fadilah N.I.M., Maarof M., Motta A., Tabata Y., Fauzi M.B. (2022). The Discovery and Development of Natural-Based Biomaterials with Demonstrated Wound Healing Properties: A Reliable Approach in Clinical Trials. Biomedicines.

[6] Wilkinson H.N., Hardman M.J. (2020). Wound healing: Cellular mechanisms and pathological outcomes. Open Biol..

[7] Rodrigues M., Kosaric N., Bonham C.A., Gurtner G.C. (2018). Wound Healing: A Cellular Perspective. Physiol. Rev..

[8] Pereira R.F., Sousa A., Barrias C.C., Bayat A., Granja P.L., Bártolo P.J. (2017). Advances in bioprinted cell-laden hydrogels for skin tissue engineering. Biomanufacturing Rev..

[9] Deng H., Li B., Shen Q., Zhang C., Kuang L., Chen R., Wang S., Ma Z., Li G. (2023). Mechanisms of diabetic foot ulceration: A review. J. Diabetes.

[10] Krause M., De Vito G. (2023). Type 1 and type 2 diabetes mellitus: Commonalities, differences and the importance of exercise and nutrition. Nutrients.

[11] Lucier J., Weinstock R.S. (2023). Diabetes Mellitus Type 1. Nih.gov.

[12] Goyal R., Jialal I., Singhal M. (2023). Type 2 Diabetes. National Center for Biotechnology Information.

[13] Bai Q., Han K., Dong K., Zheng C., Zhang Y., Long Q., Lu T. (2020). Potential Applications of Nanomaterials and Technology for Diabetic Wound Healing. Int. J. Nanomed..

[14] Burgess J.L., Wyant W.A., Abdo Abujamra B., Kirsner R.S., Jozic I. (2021). Diabetic Wound-Healing Science. Medicina.

[15] Raja J.M., Maturana M.A., Kayali S., Khouzam A., Efeovbokhan N. (2023). Diabetic foot ulcer: A comprehensive review of pathophysiology and management modalities. World J. Clin. Cases.

[16] Boyko E.J., Ahroni J.H., Stensel V., Forsberg R.C., Davignon D.R., Smith D.G. (1999). A prospective study of risk factors for diabetic foot ulcer. The Seattle Diabetic Foot Study. Diabetes Care.

[17] Jayasuriya R., Dhamodharan U., Karan A.N., Anandharaj A., Rajesh K., Ramkumar K.M. (2020). Role of Nrf2 in MALAT1/ HIF-1α loop on the regulation of angiogenesis in diabetic foot ulcer. Free Radic. Biol. Med..

[18] Alexiadou K., Doupis J. (2012). Management of Diabetic Foot Ulcers. Diabetes Ther..

[19] Rehman Z.U., Khan J., Noordin S. (2023). Diabetic foot ulcers: Contemporary assessment and management. J. Pak. Med. Assoc..

[20] Felgueiras H.P. (2023). Emerging Antimicrobial and Immunomodulatory Fiber-Based Scaffolding Systems for Treating Diabetic Foot Ulcers. Pharmaceutics.

[21] Güiza-Argüello V.R., Solarte-David V.A., Pinzón-Mora A.V., Ávila-Quiroga J.E., Becerra-Bayona S.M. (2022). Current Advances in the Development of Hydrogel-Based Wound Dressings for Diabetic Foot Ulcer Treatment. Polymers.

[22] Xu Y., Hu Q., Wei Z., Ou Y., Cao Y., Zhou H., Wang M., Yu K., Liang B. (2023). Advanced polymer hydrogels that promote diabetic ulcer healing: Mechanisms, classifications, and medical applications. Biomater. Res..

[23] Oyebode O.A., Jere S.W., Houreld N.N. (2023). Current Therapeutic Modalities for the Management of Chronic Diabetic Wounds of the Foot. J. Diabetes Res..

[24] Liu Z., Lu L. (2024). New Advances in the Treatment of Diabetic Wounds. Int. J. Biol. Life Sci..

[25] Hu Y., Li H., Lv X., Xu Y., Xie Y., Yuwen L., Song Y., Li S., Shao J., Yang D. (2022). Stimuli-responsive therapeutic systems for the treatment of diabetic infected wounds. Nanoscale.

[26] Jiang J., Li X., Li H., Lv X., Xu Y., Hu Y., Song Y., Shao J., Li S., Yang D. (2023). Recent progress in nanozymes for the treatment of diabetic wounds. J. Mater. Chem. B.

[27] Da Silva J., Leal E.C., Carvalho E., Silva E.A. (2023). Innovative Functional Biomaterials as Therapeutic Wound Dressings for Chronic Diabetic Foot Ulcers. Int. J. Mol. Sci..

[28] Yang J., Qin P., Chen Z. (2024). Application and prospect of hydrogels in diabetic wound treatment. arXiv.

[29] Wang Z., Zhao F., Deng J., Song H., Ma F., Chen L., Wang W., Xing J. (2024). Photopolymerized Multifunctional Hydrogel Loaded with Asiaticoside and Growth Factor for Accelerating Diabetic Wound Healing. ACS Appl. Polym. Mater..

[30] Bolumbu G., Mitha K. (2023). Centella asiatica and Protection in Neurodevelopment.

[31] Fira N., Auliyah R., Rahmawati A.U., Rahmawati A. (2022). Effect of Ethanol Solution Concentration in the Extraction Process of *Centella asiatica* L. Bioactive Components Using Microwave-Assisted Extraction (MAE) Method. J. Biobased Chem..

[32] Orhan I.E. (2012). *Centella asiatica* (L.) Urban: From Traditional Medicine to Modern Medicine with Neuroprotective Potential. Evid.-Based Complement. Altern. Med..

[33] Wankhade A.M., Rahangdale P.C. (2023). A Review on *Centella asiatica:* A Potential Herbal Cure. Res. J. Pharmacogn. Phytochem..

[34] Eom H.-J., Shin H.Y., Park H.J., Kim K.H., Kim J.-H., Yu K.-W. (2022). Functional components and physiological activity in different parts of *Centella asiatica*. Korean J. Food Preserv..

[35] Setiawati A., Maharani B.A., Sari P.A.P., Widyantara K.A., Saputra B.W., Febriansah R., Dwiastuti R. (2024). Deciphering the molecular pathway of an asiaticosiderich fraction of *Centella asiatica* as an anti-melanogenesis agent. J. Herbmed Pharmacol..

[36] Lim J., Lee H., Hong S., Lee J., Kim Y. (2024). Comparison of the Antioxidant Potency of Four Triterpenes of *Centella asiatica* against Oxidative Stress. Antioxidants.

[37] Bandopadhyay S., Mandal S., Ghorai M., Jha N.K., Kumar M., Radha A., Ghosh A., Proćków J., Pérez de la Lastra J.M., Dey A. (2023). Therapeutic properties and pharmacological activities of asiaticoside and madecassoside: A review. J. Cell. Mol. Med..

[38] Kumar M., Kumar D., Mahmood S., Singh V., Chopra S., Hilles A.R., Bhatia A. (2024). Nanotechnology-driven wound healing potential of asiaticoside: A comprehensive review. RSC Pharm..

[39] Min D., Yu Y., Kim T., Kim H., Lee S. (2024). Pharmacological effects of pentacyclic triterpenoids isolated from *Centella asiatica*. Hortic. Environ. Biotechnol..

[40] He Z., Hu Y., Niu Z., Zhong K., Liu T., Yang M., Ji L., Hu W. (2022). A review of pharmacokinetic and pharmacological properties of asiaticoside, a major active constituent of *Centella asiatica* (L.) Urb. J. Ethnopharmacol..

[41] Kumar M., Uttam Kumar M. (2021). Asiaticoside: A Wonderful Herbal Component of Versatile Therapeutic Benefits with Special Reference to Wound Healing Activity. J. Clin. Exp. Dermatol. Res..

[42] Tang S., Xie X., Wang M., Yang L., Wei W. (2022). Protective effects of asiaticoside on renal ischemia reperfusion injury in vivo and in vitro. Bioengineered.

[43] Li J., Zhang H., Yang X., Tan Q. (2022). Recovery of Diabetic Ulcers Facilitated by Asiaticoside through Activating the Wnt/Beta-Catenin Signalling Cascade. Indian J. Pharm. Sci..

[44] Li J., Zhang H., Tan Q. (2022). Asiaticoside expedites recovery of diabetic ulcers through activation of Wnt1/β-catenin signaling cascade. Research Square.

[45] Nie X., Zhang H., Shi X., Zhao J., Chen Y., Wu F., Yang J., Li X. (2019). Asiaticoside nitric oxide gel accelerates diabetic cutaneous ulcers healing by activating Wnt/β-catenin signaling pathway. Int. Immunopharmacol..

[46] Suriyah W.H., Rizal A.J., Nadzirin H.S.M., Ichwan S.J.A., Isa M.L.M. (2021). In Vitro Wound Healing Effect of Asiaticoside Extracted from *Centella asiatica* (‘Pegaga’) on Human Gingival Fibroblast Cell Line. Mater. Sci. Forum.

[47] Fadilah N.I.M., Phang S.J., Kamaruzaman N., Salleh A., Zawani M., Sanyal A., Maarof M., Fauzi M.B. (2023). Antioxidant Biomaterials in Cutaneous Wound Healing and Tissue Regeneration: A Critical Review. Antioxidants.

[48] Ravoor J., Thangavel M., S R.E. (2021). Comprehensive Review on Design and Manufacturing of Bioscaffolds for bone Reconstruction. ACS Appl. Bio Mater..

[49] Dong H., Zhang W., Zhou S., Huang J., Wang P. (2021). Engineering bioscaffolds for enzyme assembly. Biotechnol. Adv..

[50] Negut I., Dorcioman G., Grumezescu V. (2020). Scaffolds for Wound Healing Applications. Polymers.

[51] Sharma P., Kumar A., Agarwal T., Dey A.D., Moghaddam F.D., Rahimmanesh I., Ghovvati M., Yousefiasl S., Borzacchiello A., Mohammadi A. (2022). Nucleic acid-based therapeutics for dermal wound healing. Int. J. Biol. Macromol..

[52] Qin J., Chen F., Wu P., Sun G. (2022). Recent Advances in Bioengineered Scaffolds for Cutaneous Wound Healing. Front. Bioeng. Biotechnol..

[53] Del Bakhshayesh A.R., Annabi N., Khalilov R., Akbarzadeh A., Samiei M., Alizadeh E., Alizadeh-Ghodsi M., Davaran S., Montaseri A. (2017). Recent advances on biomedical applications of scaffolds in wound healing and dermal tissue engineering. Artif. Cells Nanomed. Biotechnol..

[54] Varghese M.G., Thomas A., Rupesh S., Sameer K.M., Joseph D., Aby Mathew T., Thomas N.G. (2024). Fabrication Techniques for Scaffolds Applied in Regenerative Medicine. Novel Biomaterials for Tissue Engineering.

[55] Hoque M.E., Chuan Y.L., Pashby I. (2011). Extrusion based rapid prototyping technique: An advanced platform for tissue engineering scaffold fabrication. Biopolymers.

[56] Adel I.M., ElMeligy M.F., Elkasabgy N.A. (2022). Conventional and Recent Trends of scaffolds fabrication: A superior mode for tissue engineering. Pharmaceutics.

[57] Dávila J.L., Freitas M.S., Neto P.I., Silveira Z.C., Silva J.V.L., D’ávila M.A. (2015). Fabrication of PCL/β-TCP scaffolds by 3D mini-screw extrusion printing. J. Appl. Polym. Sci..

[58] Jun I., Han H., Edwards J., Jeon H. (2018). Electrospun Fibrous Scaffolds for Tissue Engineering: Viewpoints on architecture and Fabrication. Int. J. Mol. Sci..

[59] Bryant S.J., Anseth K.S. (2001). The effects of scaffold thickness on tissue engineered cartilage in photocrosslinked poly(ethylene oxide) hydrogels. Biomaterials.

[60] Fadilah N.I.M., Riha S.M., Mazlan Z., Wen A.P.Y., Hao L.Q., Joseph B., Maarof M., Thomas S., Motta A., Fauzi M.B. (2023). Functionalised-biomatrix for wound healing and cutaneous regeneration: Future impactful medical products in clinical translation and precision medicine. Front. Bioeng. Biotechnol..

[61] Anand S., Rajinikanth P.S., Arya D.K., Pandey P., Gupta R.K., Sankhwar R., Chidambaram K. (2022). Multifunctional Biomimetic Nanofibrous Scaffold Loaded with Asiaticoside for Rapid Diabetic Wound Healing. Pharmaceutics.

[62] Jiang H., Zhou X., Chen L. (2022). Asiaticoside delays senescence and attenuate generation of ROS in UV-exposure cells through regulates TGF-β1/Smad pathway. Exp. Ther. Med..

[63] Witkowska K., Paczkowska-Walendowska M., Plech T., Szymanowska D., Michniak-Kohn B., Cielecka-Piontek J. (2023). Chitosan-Based Hydrogels for Controlled Delivery of Asiaticoside-Rich *Centella asiatica* Extracts with Wound Healing Potential. Int. J. Mol. Sci..

[64] Naseem M.A., Hussain Z., Thu H.E., Khan S., Sohail M., Sarfraz R.M., Mahmood A. (2024). Nanotechnology-mediated developments for improving physicochemical properties and wound healing efficacy of curcumin: A review. Int. J. Polym. Mater..

[65] Ghude P.K., Kandalkar V.P., Bhoir T.K., Nimase S.S., Bhoir P.G. (2024). Wound-healing effects of curcumin and its nano formulation. IP Int. J. Compr. Adv. Pharmacol..

[66] Elkhateeb O., Badawy M.E.I., Tohamy H.G., Abou-Ahmed H., El-Kammar M., Elkhenany H. (2023). Curcumin-infused nanostructured lipid carriers: A promising strategy for enhancing skin regeneration and combating microbial infection. BMC Vet. Res..

[67] Teerapipattanapong P., Jaikon P., Ningsanonda N., Yonemochi E., Furuishi T., Hirun N., Kraisit P. (2024). Evaluating Various Lactose Types as Solid Carriers for Improving Curcumin Solubility in Solid Self-Nanoemulsifying Drug Delivery Systems (S-SNEDDSs) for Oral Administration. Sci.

[68] Feng L., Liu Y., Chen Y., Xiang Q., Huang Y., Liu Z., Xue W., Guo R. (2023). Injectable Antibacterial Hydrogel with Asiaticoside-Loaded Liposomes and Ultrafine Silver Nanosilver Particles Promotes Healing of Burn-Infected Wounds. Adv. Healthc. Mater..

[69] Chen X., Zhang H., Liang Y., Lu Y., Xie X., Tu J., Ba L., Zhang X., Liu H. (2023). Inflammation-modulating antibacterial hydrogel sustained release asiaticoside for infection wound healing. Biomater. Adv..

[70] Zulkifli M.Z.A., Nordin D., Shaari N., Kamarudin S.K. (2023). Overview of electrospinning for tissue engineering applications. Polymers.

[71] Afjoul H., Shamloo A., Kamali A. (2020). Freeze-gelled alginate/gelatin scaffolds for wound healing applications: An in vitro, in vivo study. Mater. Sci. Eng. C.

[72] Choipang C., Buntum T., Chuysinuan P., Techasakul S., Supaphol P., Suwantong O. (2020). Gelatin scaffolds loaded with asiaticoside/2-hydroxypropyl-β-cyclodextrin complex for use as wound dressings. Polym. Adv. Technol..

[73] Bhushan S., Singh S., Maiti T.K., Sharma C., Dutt D., Sharma S., Li C., Eldin E.M.T. (2022). Scaffold Fabrication Techniques of biomaterials for bone tissue Engineering: A Critical review. Bioengineering.

[74] Narisepalli S., Salunkhe S.A., Chitkara D., Mittal A. (2022). Asiaticoside polymeric nanoparticles for effective diabetic wound healing through increased collagen biosynthesis: In-vitro and in-vivo evaluation. Int. J. Pharm..

[75] Wang P., Wang Y., Yi Y., Gong Y., Ji H., Gan Y., Xie F., Fan J., Wang X. (2022). MXenes-integrated microneedle combined with asiaticoside to penetrate the cuticle for treatment of diabetic foot ulcer. J. Nanobiotechnol..

[76] Cai Y., Xu X., Wu M., Liu J., Feng J., Zhang J. (2023). Multifunctional zwitterionic microneedle dressings for accelerated healing of chronic infected wounds in diabetic rat models. Biomater. Sci..

[77] Liang C., Wang R., He T., Chen D., Zhang G., Yin X., Wang H., Xie J., Li Y., Chen Y. (2023). Revolutionizing diabetic wound healing: The power of microneedles. Chin. J. Plast. Reconstr. Surg..

[78] Kumar S.C.S. (2018). Solid Lipid Nanoparticles Containing Asiaticoside: Development of Topical Delivery Formulation. RGUHS J. Pharm. Sci..

[79] Zou Z., Zhang Z., Gao Y., Yuan H., Guo T., He C., Chen X. (2024). Dual-Crosslinked Antibacterial Hydrogel for Treatment of Diabetic Foot Ulcers. Macromol. Chem. Phys..

[80] Loh Q.L., Choong C. (2013). Three-Dimensional Scaffolds for Tissue Engineering Applications: Role of Porosity and Pore Size. Tissue Eng. Part B Rev..

[81] Wang X., Zhang Y., Song A., Wang H., Wu Y., Chang W., Tian B., Xu J., Dai H., Ma Q. (2024). A Printable Hydrogel Loaded with Medicinal Plant Extract for Promoting Wound Healing. Adv. Healthc. Mater..

[82] Xu R., Xia H., He W., Li Z., Zhao J., Liu B., Wang Y., Lei Q., Kong Y., Bai Y. (2016). Controlled water vapor transmission rate promotes wound-healing via wound re-epithelialization and contraction enhancement. Sci. Rep..

[83] Younis H., Khan H.U., Maheen S., Saadullah M., Shah S., Ahmad N., Alshehri S., Majrashi M.A.A., Alsalhi A., Siddique R. (2023). Fabrication, Characterization and Biomedical Evaluation of a Statistically Optimized Gelatin Scaffold Enriched with Co-Drugs Loaded into Controlled-Release Silica Nanoparticles. Molecules.

[84] Liu F., Cheng Z., Yi H. (2023). NIR light-activatable dissolving microneedle system for melanoma ablation enabled by a combination of ROS-responsive chemotherapy and phototherapy. J. Nanobiotechnol..

[85] Nokoorani Y.D., Shamloo A., Bahadoran M., Moravvej H. (2021). Fabrication and characterization of scaffolds containing different amounts of allantoin for skin tissue engineering. Sci. Rep..

[86] Campos Y., Sola F.J., Fuentes G., Quintanilla L., Almirall A., Cruz L.J., Rodríguez-Cabello J.C., Tabata Y. (2021). The Effects of Crosslinking on the Rheology and Cellular Behavior of Polymer-Based 3D-Multilayered Scaffolds for Restoring Articular Cartilage. Polymers.

[87] Das P., Manna S., Roy S., Nandi S.K., Basak P. (2023). Polymeric biomaterials-based tissue engineering for wound healing: A systemic review. Burn. Trauma.

[88] Amirrah I.N., Lokanathan Y., Zulkiflee I., Wee M.F.M.R., Motta A., Fauzi M.B. (2022). A Comprehensive Review on Collagen Type I Development of Biomaterials for Tissue Engineering: From Biosynthesis to Bioscaffold. Biomedicines.

[89] Zhang Z., Cui H. (2012). Biodegradability and Biocompatibility Study of Poly(Chitosan-g-lactic Acid) Scaffolds. Molecules.

[90] Seyhan A.A. (2019). Lost in translation: The valley of death across preclinical and clinical divide–identification of problems and overcoming obstacles. Transl. Med. Commun..

[91] Vaou N., Stavropoulou E., Voidarou C., Tsigalou C., Bezirtzoglou E. (2021). Towards Advances in Medicinal Plant Antimicrobial Activity: A Review Study on Challenges and Future Perspectives. Microorganisms.

[92] Ahmad Dar A., Sangwan P.L., Kumar A. (2020). Chromatography: An important tool for drug discovery. J. Sep. Sci..

[93] Kyriakoudi A., Spanidi E., Mourtzinos I., Gardikis K. (2021). Innovative Delivery Systems Loaded with Plant Bioactive Ingredients: Formulation Approaches and Applications. Plants.

[94] Huang J., Zhou X., Shen Y., Li H., Zhou G., Zhang W., Zhang Y., Liu W. (2019). Asiaticoside loading into polylactic-co-glycolic acid electrospun nanofibers attenuates host inflammatory response and promotes M2 macrophage polarization. J. Biomed. Mater. Res. Part A.

